# Hypertension burden in CKD: is nocturnal hypertension the primary culprit?

**DOI:** 10.1093/ckj/sfaf229

**Published:** 2025-07-17

**Authors:** Francesca Mallamaci, Claudia Torino, Giovanni Tripepi

**Affiliations:** Associazione Ipertensione Nefrologia Trapianto Renale (IPNET), c/o Nefrologia, Grande Ospedale Metropolitano, Reggio Calabria, Italy; Institute of Clinical Physiology of Reggio Calabria, Italy; Institute of Clinical Physiology of Reggio Calabria, Italy

**Keywords:** ABPM, blood pressure, CKD, dialysis, hypertension

## Abstract

Hypertension is a pervasive and progressive complication in chronic kidney disease (CKD) patients, affecting up to 90% of those in advanced stages or on dialysis. A particularly insidious aspect of this condition is nocturnal hypertension, characterized by high blood pressure (BP) during sleep and a blunted or absent nighttime BP dipping—phenomena associated with accelerated CKD progression and increased cardiovascular risk. Despite its strong prognostic significance, nocturnal hypertension remains underdiagnosed due to limited use of ambulatory BP monitoring. This narrative review explores the pathophysiological underpinnings of nocturnal hypertension in CKD, including impaired sodium handling, volume overload, autonomic dysfunction and dysregulation of the renin–angiotensin–aldosterone system. Emerging evidence highlights its associations with left ventricular hypertrophy, proteinuria, endothelial dysfunction and poor renal outcomes, emphasizing the need for comprehensive BP profiling and targeted management strategies. Current therapeutic approaches include lifestyle modifications, diuretics and antihypertensive pharmacotherapy, with growing interest in chronotherapy—the timing of medication administration to align with circadian BP rhythms. However, robust clinical data specifically guiding the treatment of nocturnal hypertension in CKD remain scarce. This review underscores the clinical importance of diagnosing and addressing nocturnal BP abnormalities and advocates for future trials focused on optimizing management strategies for this high-risk population.

## INTRODUCTION

Hypertension, a major global risk factor for cardiovascular disease, is pervasive in individuals with chronic kidney disease (CKD). Its prevalence increases with disease progression, affecting nearly 90% of patients in the late CKD stages and in dialysis [1]. Salt and water retention contribute significantly to hypertension throughout all CKD stages, with their effect becoming more pronounced in the advanced stages of the disease and in dialysis [2]. While daytime blood pressure (BP) has traditionally been the focus of hypertension management, mounting evidence suggests that nocturnal hypertension—i.e. elevated BP during sleep—plays a critical role in CKD progression and adverse cardiovascular outcomes [3]. Unlike daytime BP, which can be controlled with antihypertensive therapy, nocturnal BP is less responsive to conventional treatment strategies, making its management particularly challenging. Ambulatory BP monitoring (ABPM) is recognized as the gold standard for detecting nocturnal hypertension and identifying non-dipping patterns [4] Despite its proven efficacy, ABPM remains underutilized in routine clinical practice, resulting in a diagnostic gap that contributes to the under-recognition of nocturnal hypertension thus exposing many CKD patients to increased risk for further deterioration of kidney function and heightened cardiovascular events. Moreover, the impact of nocturnal hypertension extends beyond kidney disease progression. Nocturnal hypertension has also been linked to greater proteinuria, a key marker of kidney damage and predictor of CKD progression. Given these risks, effective strategies to make diagnosis and manage nocturnal hypertension, including chronotherapy (adjusting medication timing to target nighttime BP), lifestyle interventions and personalized treatment approaches, are crucial for improving outcomes in CKD patients.

In this narrative review, we will explore key aspects of hypertension in CKD patients: (i) the disruption of nocturnal BP dipping patterns in CKD; (ii) the pathophysiological mechanisms and contributory factors driving these alterations; (iii) recent evidence on main effects of reduced nocturnal BP burden on cardiovascular and renal health; and (iv) therapeutic approach to hypertension in CKD patients.

## DISRUPTION OF NOCTURNAL BP DIPPING PATTERN IN CKD

**Figure 1:**
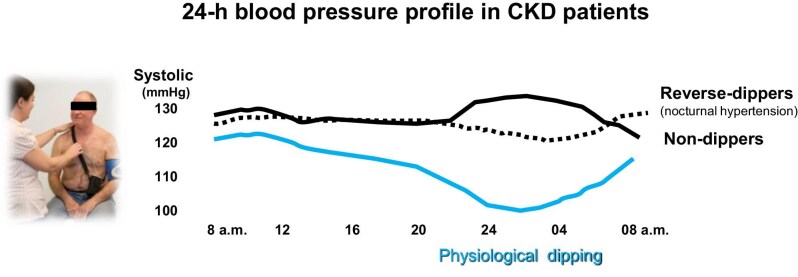
24-h blood pressure profile in CKD patients.

In patients with CKD, the introduction of 24-ABPM has revolutionized our understanding of BP dynamics, providing critical insights that were previously unknown. This method was first recommended by the National Institute for Health and Care Excellence (NICE) guidelines for diagnosing hypertension at the community level, aiming to enhance the care and management of individuals with elevated BP (https://www.nice.org.uk/guidance/ng136/chapter/recommendations). ABPM offers a comprehensive profile of BP fluctuations, capturing variations that may not be apparent during standard clinic visits. A typical 24-h BP profile demonstrates a physiological nocturnal dip, where BP decreases by approximately 10% to 20% during sleep [5]. This nocturnal decline is a vital aspect of cardiovascular health, reflecting the body's ability to regulate BP in response to circadian rhythms and autonomic nervous system (ANS) function. In CKD patients, a paradoxical increase in nocturnal BP is often observed, with nighttime levels frequently exceeding those recorded during the day [6] (Fig. 1). This phenomenon presents additional challenges for clinicians, as traditional daytime BP measurements may significantly underestimate the true hypertensive burden faced by these patients.

The failure to recognize and address nocturnal hypertension can result in inadequate management strategies, perpetuating a cycle of increased cardiovascular risk and renal disease progression. The prevalence of the absence of a nocturnal BP decline and/or nocturnal hypertension is particularly high among CKD patients, driven by factors such as volume overload, impaired sodium handling and autonomic dysfunction [7]. Additionally, sleep apnea, which is also common in CKD, significantly contributes to these alterations by inducing intermittent hypoxia, increasing sympathetic activity and finally impairing BP regulation [8]. Recognizing the multifaceted nature of nocturnal hypertension in CKD is crucial for developing effective management strategies that can mitigate its adverse effects on both kidney and cardiovascular health. Of note, the absence of a nocturnal BP decline is recognized as a significant CV risk factor. Non-dipping is correlated with an increased risk of left ventricular hypertrophy, cardiovascular events and overall mortality [9]. In CKD patients, the implications of non-dipping are compounded by the underlying pathophysiological changes associated with renal impairment, such as volume overload, sympathetic nervous system activation and dysregulation of the renin–angiotensin–aldosterone system (RAAS). Therefore, the implementation of 24-h ABPM in clinical practice is crucial for accurately identifying individuals at risk and tailoring therapeutic interventions accordingly [10].

## MECHANISMS FOR NOCTURNAL HYPERTENSION IN CKD PATIENTS

Nocturnal hypertension in individuals with CKD is a complex phenomenon driven by multiple pathophysiological mechanisms, among which impaired volume regulation is particularly significant.

CKD is marked by a progressive decline in renal function that substantially diminishes the kidneys’ capacity for effective sodium excretion. This impairment leads to sodium retention, a disturbance that becomes increasingly pronounced in advanced stages of the disease due to ongoing nephron loss and glomerulosclerosis. As the ability to eliminate excess sodium declines, extracellular fluid volume expands, resulting in volume overload. This state of fluid excess is a major contributor to elevated nocturnal BP, a time during which a physiological decline in BP—known as the nocturnal dipping pattern—is typically observed but often blunted or absent in CKD patients [11].

The inadequate excretion of sodium disrupts the homeostatic mechanisms that regulate BP, thereby facilitating the onset of nocturnal hypertension. Moreover, the retention of sodium not only contributes to increase BP levels but also exacerbates vascular stiffness and endothelial dysfunction, thereby intensifying the cardiovascular risks associated with CKD. The interplay between sodium retention and these vascular changes underscores the complexity of managing hypertension in this patient population.

**Figure 2:**
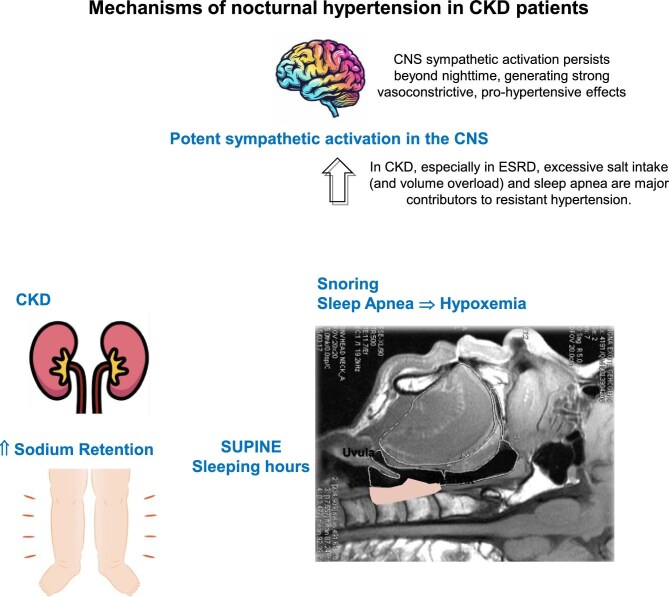
Mechanisms of nocturnal hypertension in CKD patients.

Another contributing factor to nocturnal hypertension is ANS dysfunction, common in CKD [12] (Fig. 2). The ANS plays a crucial role in the regulation of cardiovascular function, including BP homeostasis. In patients with CKD there is a notable disruption in the normal functioning of the ANS, characterized by an imbalance between sympathetic and parasympathetic activity [13]. This dysregulation manifests as increased sympathetic outflow, which is often associated with increased heart rate and vascular resistance. The loss of normal circadian rhythms in autonomic regulation further complicates BP management, leading to abnormal BP patterns during the night, further exacerbating sympathetic activation and creating a vicious cycle that perpetuates elevated nighttime BP.

Increased activity of the sympathetic nervous system (SNS) significantly contributes to the persistence of elevated nocturnal BP. The pathophysiological mechanisms underlying this increased sympathetic drive include renal ischemia, which stimulates the release of norepinephrine and other neurohormonal factors that activate the SNS [12]. Elevated sympathetic activity leads to vasoconstriction, increased heart rate and enhanced cardiac output, all of which contribute to elevated BP levels. Furthermore, the sympathetic nervous system interacts with the RAAS, creating a feedback loop that exacerbates hypertension. In CKD, the combination of renal dysfunction and increased sympathetic tone results in a failure to achieve the expected nocturnal dipping in BP, further solidifying the association between increased sympathetic activity and nocturnal hypertension.

The RAAS is another critical regulator of BP and fluid balance. In CKD, the dysregulation of the RAAS is a significant contributor to the pathophysiology of hypertension, particularly nocturnal hypertension [14]. As kidney function declines, there is often an inappropriate activation of the RAAS, leading to increased levels of renin, angiotensin II and aldosterone. Angiotensin II is a potent vasoconstrictor that increases systemic vascular resistance, while aldosterone promotes sodium retention and fluid overload. This excessive activation of RAAS not only raises BP during the day but also impairs the normal nocturnal decline in BP. The resultant fluid retention and vasoconstriction create a state of volume overload, which is particularly detrimental during the night when the body typically experiences a physiological reduction in BP. Moreover, the chronic elevation of angiotensin II levels has been associated with endothelial dysfunction and increased vascular stiffness, further complicating the management of BP in CKD patients. Therefore, the dysregulation of RAAS plays a pivotal role in the development and persistence of nocturnal hypertension in this population, highlighting the need for targeted therapeutic interventions aimed at modulating this system to improve cardiovascular outcomes.

## RECENT EVIDENCES ON MAIN EFFECTS OF REDUCED NOCTURNAL BP BURDEN ON CARDIOVASCULAR AND RENAL HEALTH IN CKD PATIENTS

Emerging research has underscored the significant impact of nocturnal BP abnormalities on cardiovascular and renal health in CKD patients. Overall, these findings suggest that nighttime BP patterns provide crucial prognostic information, with implications for both risk stratification and treatment optimization in CKD patients. Understanding the interplay between nocturnal hypertension, autonomic dysfunction and vascular health is vital for improving patient outcomes. A study published by Fu *et al*. highlighted the high prevalence of nocturnal hypertension in CKD patients with masked uncontrolled hypertension (MUCH) and its strong association with left ventricular hypertrophy (LVH) and poor renal outcomes [15]. This poses a serious risk because nocturnal hypertension has been shown to exert a stronger influence on target organ damage than daytime BP elevations. LVH, a structural abnormality of the heart characterized by thickening of the left ventricular walls, is a well-known predictor of cardiovascular morbidity and mortality. The study found that CKD patients with MUCH and nocturnal hypertension had a significantly higher frequency of LVH, suggesting that nocturnal BP abnormalities may contribute to pathological cardiac remodelling [15]. LVH is a critical concern because it increases the risk of heart failure, arrhythmias and sudden cardiac death. Thus, routine nocturnal BP monitoring may be essential for identifying at-risk patients and initiating timely therapeutic interventions to prevent adverse cardiovascular outcomes. The results of the paper by Fu *et al*. are germane to those found by Borrelli *et al*. [16] showing that systolic ambulatory BP above goal or the absence of nocturnal dipping, regardless of ambulatory BP, is associated with higher risks of cardiovascular disease and kidney disease progression among patients with CKD. Jeong *et al*. [17] examined the relationship between adverse nocturnal BP profiles, sympathetic nerve activity and endothelial dysfunction in CKD patients. The study specifically focused on patients exhibiting non-dipping or reverse-dipping BP patterns—conditions in which BP fails to decline at night or paradoxically increases. These abnormal patterns were associated with increased muscle sympathetic nerve activity, a marker of ANS dysfunction. Excessive sympathetic activation can lead to persistent vasoconstriction, increased heart rate and elevated BP, all of which contribute to cardiovascular stress. Additionally, the study identified a link between abnormal nocturnal BP profiles and endothelial dysfunction, a condition in which the inner lining of blood vessels fails to function properly. Endothelial dysfunction is a precursor of atherosclerosis and is associated with increased arterial stiffness, reduced nitric oxide availability and impaired vascular reactivity [18]. These findings suggest that nocturnal BP abnormalities may not only be a consequence of CKD but also an active driver of disease progression through sustained sympathetic activation and vascular damage. Addressing sympathetic overactivity through lifestyle modifications, pharmacological interventions or autonomic modulation therapies could be a promising strategy to mitigate cardiovascular risk in CKD patients. The BP burden during nighttime can be also expressed in terms of nighttime double product—a metric calculated as the product of nocturnal systolic BP and pulse rate. The double product serves as an indirect measure of myocardial workload and SNS activity. In a recent retrospective cohort study including a total of 1434 patients with nondialysis CKD complicated by hypertension [19], elevated nighttime double product was strongly associated with an increased risk of major cardiovascular and cerebrovascular events, all-cause mortality and worsening renal function. This highlights the importance of not only monitoring absolute BP values but also considering composite indicators that reflect cardiovascular stress. The study's findings suggest that elevated nighttime double product may serve as an early warning sign for CKD patients at heightened risk for adverse outcomes. This could have significant clinical implications, as it may help refine risk stratification models and guide the development of targeted interventions aimed at reducing nighttime BP burden and myocardial workload. Future research should explore whether therapeutic strategies, such as beta-blockers or lifestyle interventions aimed at lowering nighttime heart rate and BP, could help improve outcomes in this high-risk population. The importance of nighttime BP patterns is further reinforced by the observation that a diminished nocturnal systolic BP decline is linked to adverse kidney outcomes in CKD patients [20]. The association between nocturnal systolic BP dipping and CKD progression was examined in 1061 participants at the Cardiovascular and Metabolic Disease Etiology Research Center-High Risk (CMERC-HI). The study found that non-dipping and reverse-dipping patterns were associated with a significantly higher risk of renal function deterioration over time. In particular, patients with non-dipping and reverse dipping patterns were at higher risk for CKD progression than those with a dipping pattern. The physiological basis for this association may stem from the fact that persistent nocturnal hypertension leads to increased glomerular pressure, accelerated nephron loss and heightened renal ischemia. The kidneys rely on fluctuations in BP to maintain optimal perfusion and filtration balance; when BP remains persistently elevated at night, it can cause glomerular hyperfiltration and progressive damage to renal structures.

## THERAPEUTIC STRATEGIES

### Lifestyle modifications in hypertension management

The most recent KDIGO Guidelines on BP management in CKD patients [21] recommend that systolic BP target should be <120 mmHg, provided that this target is well tolerated by the patient. Ultimately, based on a cluster analysis derived from the SPRINT study, the Guidelines suggest a systolic BP target of <130 mmHg, which represents an optimal balance between efficacy and safety [22]. Before delving into the pharmacological management of hypertension, it is crucial to emphasize lifestyle modifications aimed at reducing BP in CKD patients. A key recommendation from the latest guidelines is to limit daily salt intake to <2 g. Additionally, engaging in some form of physical activity is also encouraged, as a sedentary lifestyle is a significant cardiovascular risk factor for both the general population and individuals with various chronic conditions. A large clinical trial in CKD patients on dialysis, documented the beneficial effect on physical performance and outcomes of physical exercise [23].

Salt intake is another important lifestyle factor that contributes to elevated BP in patients with CKD [24–26]. Diuretic therapy or dietary salt restriction can help restore the circadian BP rhythm to a dipper pattern by preventing sodium retention [25]. Fukuda *et al*. [26] also reported that as renal function declines in glomerulopathy, the normal nocturnal dipping of BP is lost. This results in increased nighttime urinary excretion of sodium and protein. They suggest that impaired natriuresis during the day leads to elevated nocturnal BP as a compensatory mechanism through pressure natriuresis. Consequently, nocturnal glomerular capillary hypertension may partly account for the increased nighttime excretion of sodium and protein.

### Pharmacological approach and use of diuretics

Following lifestyle recommendations, the subsequent focus has to be addressed to pharmacological interventions, particularly the re-evaluation of therapeutic strategies involving diuretics, specifically thiazide diuretics in CKD patients not on dialysis. Conventional hypertension management typically follows a stepwise approach, beginning with dietary sodium reduction, followed by the addition of a diuretic. This may be complemented with the introduction of angiotensin-converting enzyme inhibitors or calcium channel blockers, and subsequently, agents that modulate the sympathetic nervous system. If BP remains uncontrolled, an aldosterone antagonist may be introduced, provided potassium levels are within the normal range, treatment which often helps in managing resistant hypertension. Diuretics have been extensively studied in randomized controlled trials involving CKD patients, both as primary agents and adjunct therapies. The volume-dependent nature of hypertension in CKD underscores the importance of salt reduction in managing hypertensive patients. While dietary sodium restriction has proven to be effective in lowering BP and alleviating volume overload, adherence to such dietary changes can be challenging due to the high sodium content in many foods. Therefore, exploring alternative strategies, such as the use of diuretics, becomes imperative. A double-blind trial compared the efficacy of diuretics with low-sodium diets in reducing BP among hypertensive CKD patients not on dialysis [27]. Both interventions were successful in lowering mean 24-h systolic BP. A study in 160 patients with stage 4 CKD [estimated glomerular filtration rate (eGFR) 15–30 mL/min] [28] documented the antihypertensive potential of chlorthalidone in this population. In this study, participants with uncontrolled hypertension (confirmed through 24-h ABPM) were randomized to receive chlorthalidone (starting at 12.5 mg/day, titrated up to 50 mg/day if necessary) or a placebo for a duration of 12 weeks. At baseline, the mean eGFR was 23.2 mL/min, and the average number of antihypertensive medications prescribed was 3.4, with 60% of participants receiving loop diuretics. The source population comprised over 2000 patients, and findings indicated that chlorthalidone therapy significantly improved BP control at 12 weeks compared with placebo among patients with advanced CKD and poorly controlled hypertension.

### Chronotherapy: timing of antihypertensive medication

It is noteworthy once again to underline that while there are studies on the management of high BP during the day, the crucial question is the treatment of the most neglected and underdiagnosed aspect of nocturnal hypertension in CKD patients. Information and data on this peculiar aspect are scanty despite the fact that approximately 60% of CKD patients experience elevated nighttime BP. A pivotal study by Minutolo *et al*. [29] investigated the utility of administering antihypertensive therapy during nighttime hours in non-dipper patients. In this cohort of 32 non-dipper patients, adjustments in therapy resulted in reductions in both systolic and diastolic BP, with nearly all patients achieving the recommended BP targets. The question of whether the timing of antihypertensive drugs administration influences outcomes has been explored in many studies on hypertensive patients, although results remain controversial. A recent multicenter, controlled, prospective trial involving 19 084 hypertensive patients (10 614 men and 8470 women, mean age 60.5 ± 13.7 years) randomized participants to take their daily antihypertensive medications either at bedtime (*n* = 9552) or upon awakening (*n* = 9532) [30]. Results favoring bedtime administration were highlighted, with the authors concluding that routine ingestion of antihypertensive medications at bedtime, compared with morning dosing, leads to improved ambulatory BP control, enhanced nighttime relative BP decline and a significantly reduced incidence of major cardiovascular events. The TIME study [31] involved nearly 20 000 patients and aimed to assess the primary composite endpoint of vascular death or hospitalization due to non-fatal myocardial infarction or non-fatal stroke. The cumulative outcomes indicated no significant differences between evening and morning dosing regarding major cardiovascular outcomes, suggesting that patients may take their regular antihypertensive medications at a time that is convenient and minimizes adverse effects. Furthermore, a systematic review and meta-analysis [32] reported that evening dosing of antihypertensive medications resulted in a slight reduction in total and daytime ambulatory systolic BP (1.41 mmHg and 0.94 mmHg, respectively), with a more pronounced effect on nighttime systolic BP (4.09 mmHg). While evening dosing numerically reduced cardiovascular events compared with morning dosing, the effect diminished when controversial data from Hermida's studies (23 trials, 25 734 patients) were excluded, leading to no significant impact on 24- or 48-h ambulatory systolic BP, daytime BP, nighttime BP or major adverse cardiac events. While daytime BP management is well-established, the importance of nighttime BP control is restlessly gaining recognition. The timing of antihypertensive medications could influence nighttime BP. For example, administering certain medications in the evening may help reduce nighttime hypertension. Medications like long-acting calcium channel blockers or certain diuretics can be beneficial when taken at night. Yet there is little or no knowledge whether timing hypertension treatment could be beneficial on outcomes.

When discussing BP medications, it is important to consider the findings of a recent Cochrane meta-analysis [33]. This analysis showed that evening administration of once-daily antihypertensive drugs resulted in a modest reduction in 24-h BP, without significantly affecting daytime values. On average, nocturnal systolic BP was approximately 2 mmHg lower when medications were taken at bedtime compared with morning dosing. However, chronotherapy may not be effective in all patient populations. For example, among Black patients with hypertensive CKD, neither bedtime dosing of once-daily antihypertensives nor the addition of nighttime medications significantly reduced nocturnal BP compared with morning administration [33].

Moreover, tailoring treatment for each patient with CKD and hypertension could be a further step in this category of patients. Finally, regular follow-up and monitoring of both daytime and nighttime BP are crucial. Adjustments to therapy should be made based on comprehensive BP profiles rather than solely relying on office readings. It is worth stating that further research is needed to establish optimal treatment strategies and guidelines specifically addressing nighttime hypertension in CKD patients. For instance, chronotherapy, which involves administering antihypertensive medications at specific times to align with the body's circadian rhythms, has shown promise in improving nocturnal BP control. Additionally, lifestyle modifications, such as dietary sodium restriction and weight management, may also play a role in mitigating nocturnal hypertension. But all these “opinions” have to be translated to “facts,” and to this aim, specifically designed clinical trials in non-dipper or with nocturnal hypertension CKD patients would be welcome.

## CONCLUSIONS

In summary, 24-h ABPM serves as an invaluable tool in the management of hypertension in patients with CKD. The disruption of the physiological nocturnal BP drop, leading to non-dipping patterns and paradoxical nocturnal hypertension, underscores the importance of comprehensive BP assessment. Recognizing these patterns is essential for identifying cardiovascular risk and implementing effective management strategies.

Future and ongoing (https://www.clinicaltrials.gov/study/NCT02990663) research will better explore the implications of nocturnal hypertension in CKD and evaluate new treatment approaches to restore normal BP patterns, ultimately improving patient outcomes and reducing cardiovascular morbidity and mortality in this vulnerable population.

However, unless the primary objective is to specifically reduce nighttime BP, antihypertensive medications should be administered at a time that is most convenient for the patient, thereby optimizing adherence and minimizing undesirable side effects.

## Data Availability

The data underlying this article are available in the article and in its online supplementary material.
